# An Online Data Visualization Feedback Protocol for Motor Imagery-Based BCI Training

**DOI:** 10.3389/fnhum.2021.625983

**Published:** 2021-06-07

**Authors:** Xu Duan, Songyun Xie, Xinzhou Xie, Klaus Obermayer, Yujie Cui, Zhenzhen Wang

**Affiliations:** ^1^School of Electronics and Information, Northwestern Polytechnical University, Xi’an, China; ^2^Faculty of Electrical Engineering and Computer Science, Technical University Berlin, Berlin, Germany

**Keywords:** brain–computer interface, motor imagery, training protocol, feedback, Riemannian geometry

## Abstract

Brain–computer interface (BCI) has developed rapidly over the past two decades, mainly due to advancements in machine learning. Subjects must learn to modulate their brain activities to ensure a successful BCI. Feedback training is a practical approach to this learning process; however, the commonly used classifier-dependent approaches have inherent limitations such as the need for calibration and a lack of continuous feedback over long periods of time. This paper proposes an online data visualization feedback protocol that intuitively reflects the EEG distribution in Riemannian geometry in real time. Rather than learning a hyperplane, the Riemannian geometry formulation allows iterative learning of prototypical covariance matrices that are translated into visualized feedback through diffusion map process. Ten subjects were recruited for MI-BCI (motor imagery-BCI) training experiments. The subjects learned to modulate their sensorimotor rhythm to centralize the points within one category and to separate points belonging to different categories. The results show favorable overall training effects in terms of the class distinctiveness and EEG feature discriminancy over a 3-day training with 30% learners. A steadily increased class distinctiveness in the last three sessions suggests that the advanced training protocol is effective. The optimal frequency band was consistent during the 3-day training, and the difference between subjects with good or low MI-BCI performance could be clearly observed. We believe that the proposed feedback protocol has promising application prospect.

## Introduction

Brain–computer interface (BCI) is an innovative control method that functions independently of the human peripheral nerves and muscles (Wolpaw et al., 2000; Ramadan and Vasilakos, 2017). BCI technology has developed rapidly over the past 20 years, mainly due to the introduction of machine learning methods that improve the accuracy and speed of BCI pattern recognition. Subjects must learn to modulate their brain signals to ensure a reliable BCI, which can be problematic. BCI skill corresponds to the subject’s ability to voluntarily produce brain activity patterns that are distinct among different mental tasks and stable within one mental task, so that they can be translated reliably and consistently into control commands (Lotte and Jeunet, 2018). However, researchers have tended to focus on the machine learning aspects of BCI training. If subjects are unable to generate distinguishable electroencephalogram (EEG) patterns, it is difficult for a decoding algorithm to recognize them. Studies have suggested that the nature of subject learning in MI-BCI (motor imagery-BCI) mostly belongs to an implicit process (Kober et al., 2013; Perdikis and Millan, 2020). “Implicit” learning suggests that subjects acquire some BCI skills imperceptibly and gradually with the help of feedback observation, which cannot be passed on to others verbally or schematically (Sitaram et al., 2017). Thus, feedback training has been proven to be a practical approach to MI-BCI subject learning (Neuper and Pfurtscheller, 2010). Implicit learning may be better facilitated by more natural feedback provision strategies than explanatory or instructional feedback (Schumacher et al., 2015; Corbet et al., 2018).

Arrouët et al. (2005) visualized brain activation feedback as a three-dimensional pattern of intra-cranial current density. Hwang et al. (2009) created a real-time topography of cortical activation at the feedback session. These two studies, indeed, provided the visualization of some neurophysiological activities directly to the subjects, which falls under the category of neurofeedback approach to learning. Conversely, the modern BCI training system is driven by classification outputs that combine several spatio-spectro-temporal features of brain activity to enable simpler, neurofeedback-like visualizations. Non-invasive BCI training may consist of asking subjects to perform mental imagery tasks, such as the kinesthetic motor imagery (MI) of body limbs by imagining left- or right-hand movements to move a cursor on a screen in different directions depending on the mental task recognized by a classifier. Such tasks may be combined with some form of classification certainty, such as the probability or the distance from a separation hyperplane (Blankertz et al., 2007; Chavarriaga et al., 2017). Kaufmann et al. (2011) provided BCI users with a cursor and asked them to target not only its direction but also its color and intensity. Sollfrank et al. (2016) designed a liquid floating through a visualization of a funnel to gather enriched visual feedback, including information regarding EEG uncertainty. However, showing all features to the subject is not particularly attractive as it is believed that subjects will be overwhelmed by such complexity.

Most training feedback approaches are dependent on classification results, which creates significant limitations. The training of a given classifier, for example, requires the identification of the target state from a previous calibration recording, which is overly time-consuming. Researchers have investigated automatic calibration approaches, but their subjects still had to face an unintuitive and tedious period of lacking feedback in completing their tasks (Faller et al., 2012, 2014; Kus et al., 2012). Many approaches provide continuous feedback signals within a trial range (a few seconds) before the feedback image is cleared during the interval and re-presented in the subsequent trial (Acqualagna et al., 2016; Sollfrank et al., 2016; Penaloza et al., 2018). Inexperienced subjects may subconsciously employ different mental strategies over time, so feedback signals should be gradually accumulated and kept on the screen over a more extended period. We hypothesize that subjects will learn more efficiently when able to compare prior trials with current trials while observing non-reset feedback.

To this end, we propose measuring the dissimilarities between respective brain patterns and visualizing them as feedback from the very beginning to the end of the learning session. The pairwise distances of all EEG measurements in a long period can be converted into the coordinate position on a 2D screen and gradually presented to the subjects without any calibration period. The distances, rather than classification results, could provide a more precise description of the subject’s current EEG pattern over the entire run. The spatial covariance, as a symmetric positive definite (SPD) matrix, is commonly used to parametrize multi-channel EEG information in a specific frequency band. SPD matrices are defined in a Riemannian manifold. Accordingly, operations involving them should respect the intrinsic geometry of this manifold. Congedo et al. (2017) introduced Riemannian geometry to EEG classifications such as Fisher Geodesic Discriminant Analysis, Riemannian kernel-SVM, and transfer learning (Barachant et al., 2012, 2013b; Zanini et al., 2018). Lotte and Jeunet (2018) proposed metrics based on Riemannian geometry to quantify the subjects’ BCI performance independently of any classifier. Riemannian geometry methods have not yet been used for BCI training. In this study, the Riemannian distances among the spatial covariances of multi-channel EEGs were measured and non-linearly transmitted to subjects. Feedback points on the screen were used as a concise representation of EEG measurements, where the relative positions of points denote the similarities among them. Subjects learned to separate points of two categories and to centralize points within the same category. We also monitored the quality of the EEG segment and abandoned the artifacts in real time.

The purpose of this work is to determine whether the proposed feedback protocol is an effective way to improve subjects’ MI-BCI skills. We quantified the subjects’ performance and learning skills, including their competency in the training process and ability to upgrade their skills over the course of the entire experiment. We gathered the neurophysiological evidence of subject learning and explored the optimal EEG frequency band for each subject during the training sessions. The feedback protocol may be useful for subjects who attempt to improve their MI-BCI skills to accomplish BCI applications. However, due to the uncontrolled experimental design, we could not prove that the proposed approach is superior to the conventional cursor feedback training.

## Materials and Methods

Ten healthy subjects were recruited to participate in the proposed MI-BCI training experiment. Five are female. The average age among them was 23.4 ± 3.69 years, ranging from 20 to 33 years of age. As shown in Table 1, 4 out of 10 subjects had former BCI experience. Specifically, s4, s5, and s9 have performed SSVEP-BCI experiment for half an hour, and s7 has performed MI-BCI experiment for two sessions (about 1 h). We also investigated subjects’ handedness according to the Chinese Handedness Scale (Li, 1983), which includes writing, holding chopsticks, throwing things, brushing teeth, using scissors, lighting a match, threading a needle, holding a hammer, holding a racket, and washing their face with a towel. If a subject gets used to doing all 10 items with the left hand dominant, he or she was considered to have “strong left-handedness.” A subject had “left-handedness,” if he or she gets used to doing only the first six items with the left hand and any one of the remaining four items with the right hand, and *vice versa*. If a subject gets used to doing one to five items among the first six with one hand and the remaining items with the other hand, he or she was considered to have “mixed handedness.” As shown in Table 1 (Column 3), the number of subjects with “strong right-handedness,” “right-handedness,” and “mixed handedness” are 7, 2, and 1, respectively. None were left-handed.

**TABLE 1:** The former BCI experience, handedness, MI-BCI performance, and the optimal EEG frequency band for 10 subjects.

Subject number	Former BCI experience	Handedness	MI-BCI performance	Optimal frequency band (Hz)	
	
				First two sessions	All six sessions
s1	No	Strong-right	“Good”	9–13	8–12, 9–13
s2	No	Mixed	“Low”	26–30	12–16, 26–30
s3	No	Strong-right	“Low”	9–13	9–13, 8–12
s4	SSVEP-BCI	Mixed	“Low”	19–23	19–23, 18–22
s5	SSVEP-BCI	Strong-right	“Low”	26–30	26–30, 25–29
s6	No	Right	“Low”	26–30	26–30, 13–17
s7	MI-BCI	Strong-right	“Good”	10–14	10–14, 11–15
s8	No	Strong-right	“Low”	26–30	26–30, 25–29
s9	SSVEP-BCI	Strong-right	“Good”	11–15	11–15, 10–14
s10	No	Strong-right	“Low”	26–30	26–30, 25–29

*The MI-BCI performance is measured in the first session. If the *ClassDis* of the first session was higher than the average *ClassDis* among 10 subjects, the subject was considered to have “good” MI-BCI performance; otherwise, they were treated as having “low” MI-BCI performance.*

### Experimental Setup

**FIGURE 1:**
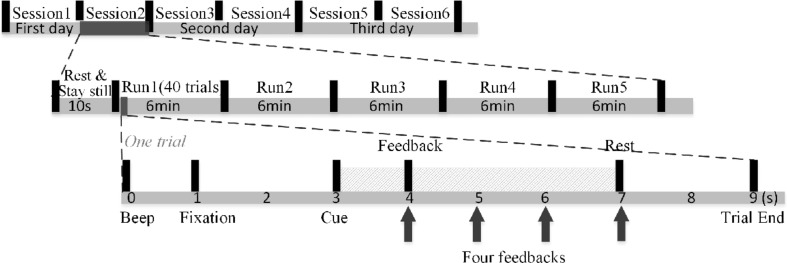
MI-BCI training structure.

As shown in Figure 1, MI-BCI training was conducted for three consecutive days. Each day contained two sessions, for a total of six sessions. Subjects were allowed to rest for any amount of time at will, and the EEG cap was not removed or replaced between two sessions on the same day. Five runs, each of approximately 6-min duration, make up one session. In each session, subjects were asked to rest and remain still for 10 s before the first run. The 10-s resting-state EEG measurement was considered a baseline for the initialization of the artifact detection. Subjects were free to rest as long as they wished in the interval between two runs. Each run was composed of 40 trials. At the trial start, a “beep” sound prompted subjects to shift their attention from the feedback on the right-side screen to the instruction on the left-side screen. The fixation appeared to remind subjects to stay still. After 2 s, the subjects performed MI of the left hand as the left arrow appeared (with an auditory “left” reminder synchronously), or the right hand when the right arrow appeared (accompanied by an auditory “right” reminder). The MI task lasted 4 s, during which the feedback was updated at the end of the first, second, third, and fourth seconds. A 2-s rest period was then offered and the next trial proceeded. The subjects were instructed to suppress muscle movement to prevent artifacts. All experimental procedures were approved by Northwestern Polytechnical University Medical and Experimental Animal Ethics Committee with written informed consent from all subjects.

A 10–20 system EEG cap and Neuracle wireless amplifier were used to record the EEG signals. EEGs from 30 electrodes (F3, FZ, F4, FT7, FC5, FC3, FC1, FCz, FC2, FC4, FC6, FT8, C5, C3, C1, Cz, C2, C4, C6, CP3, CP1, CP2, CP4, P5, P3, PZ, P4, P6, PO7, and PO8) distributed symmetrically over two hemispheres of the sensorimotor area were collected during the training experiment. Only 10 electrodes (FC3, FC4, C5, C3, C1, C2, C4, C6, Cp3, and Cp4) were employed for feedback. The reference electrode Cz was placed in the central–parietal area. All impedances were kept below 5 kΩ. The EEG signals were band-pass filtered between 0.01 and 100 Hz and sampled at 250 Hz.

### Experimental Design and Protocol

The 3-day training experiments were carried out under the same feedback protocol apart from an optimal subject-specific frequency band that was employed for the last 2 days. Subjects were seated in a comfortable chair in front of a computer screen in a quiet laboratory. The screen was divided into left and right parts, as mentioned above. Subjects were given instructions on the left side of the screen and feedback on the right. EEG measurements were taken in four 1-s segments using a 1-s window with zero overlaps during the 4-s feedback period from when the arrow appeared to the end of the hand imagination in each trial. At the end of the first, second, third, and fourth seconds, the 1-s EEG measurement was used to display an updated five-step feedback point. The current 1-s EEG measurement was judged as an artifact or not with the Riemannian Potato method. If the EEG was of good quality, the EEG was retained and the following four steps proceeded. If not, the EEG was abandoned and a sharp beep was emitted to remind the subject to stay still and concentrate.

Next, the retained 1-s EEG was band-pass filtered in 8–30 Hz for the first two sessions and in a narrower subject-specific 4-Hz wide frequency band for the rest of the sessions. The covariance matrix for the filtered 10-channel EEG was estimated. All previous 1-s EEG covariance matrices that belonged to the feedback period within the current run were saved, the number of which was assumed as *N*. The pairwise Riemannian distances among *N* trials of EEG covariance matrices were calculated to create an *N* × *N* symmetric matrix. In order to display each 1-s EEG as a point on the 2D screen, the distance matrix was reduced into two dimensions with diffusion map (Coifman et al., 2005). The diffusion map is a non-linear dimension reduction tool that integrates local similarities to provide a global description of samples.

**FIGURE 2:**
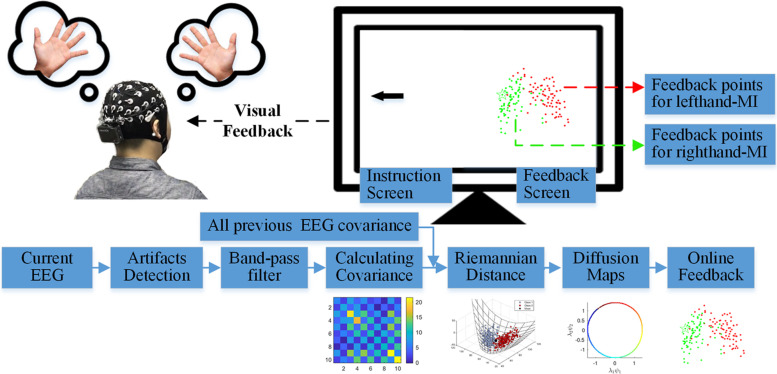
MI-BCI training feedback protocol.

Finally, the 2D vector was plotted in a coordinate axis across a scatter diagram, as shown in Figure 2 and Supplementary Video 1. The red and green points represent 1-s EEG measurements for the left-hand MI and right-hand MI, respectively, and the red and green rectangles denote the center of mass for the two respective conditions. The updated point is marked with a five-pointed star with the color of the corresponding category. The distance between the two rectangles represents the dissimilarity between two tasks from all prior EEG measurements. When a point falls on the periphery of the point cluster (but not beyond a specific range), the recent mental activity differs from other mental states. When a point falls near the overlap of the two-point clusters, conversely, the current mental activity is relatively similar to another mental state.

### Online Signal Processing

EEG signals were subjected to online data preprocessing (artifact detection), feature extraction (Riemannian geometry), and the generation of feedback (diffusion map). Traditionally, the SPD matrices are treated as if they are naturally lying in Euclidean space, whereas the natural geometry to be considered is Riemannian. We utilized several essential tools that have been introduced in Barachant et al. (2012) and Congedo et al. (2017) to manipulate the EEG data in a Riemannian manifold, and employed a manifold learning, diffusion map, to realize the visualization of feedback points.

#### Riemannian Geometry Methods

We registered the 1-s band-pass filtered EEG measurement as **X** ∈ *ℝ*^*n* × *T*^, where *n* and *T* denote the number of channels and the sample points, respectively (*n* = 10, *T* = 250 in this experiment). The *n* * *n* symmetric covariance matrix of each EEG measurement can be defined as

(1) $${\text{P}}_{X}=\frac{1}{T-1}\,{\text{XX}}^{T}$$

The basic properties of SPD matrix spaces can be found in the literature (Congedo et al., 2017). We set 𝒫(*n*) as the space of the set of SPD matrices. The distance between two points **P**_1_,**P**_2_ ∈ 𝒫(*n*), namely, the geodesic connection between **P**_1_ and **P**_2_, can be expressed as

(2) $$\delta \,\left({\text{P}}_{1},{\text{P}}_{2}\right)={||\log\left({\text{P}}_{1}^{-1/2}\,{\text{P}}_{2}\,{\text{P}}_{1}^{-1/2}\right)||}_{F}={\left(\sum_{i=1}^{n}\log^{2}\lambda_{i}\right)}^{1/2}$$

where ||⋅||_*F*_ denotes the Frobenius norm and λ_*i*_,*i* = 1,…*n* denotes the eigenvalues of ${\text{P}}_{1}^{-1}\,{\text{P}}_{2}$. Two statistical descriptors, the Riemannian mean $\bar{\text{P}}$ and the mean absolute deviation σ_*P*_, of a set of SPD matrices are:

(3) $$\bar{\text{P}}={\text{argmin}}_{P}\,\sum_{i=1}^{N}\delta_{R}^{2}\,\left({\text{P}}_{i},P\right)$$

The Riemannian mean of *N* SPD matrices (*N* > 2) serve to find a point in the SPD manifold with the nearest distance to each SPD matrix. The Riemannian mean $\bar{\text{P}}$ can be iteratively identified in the literature (Moakher, 2005). We set the first and second experimental trials as two different categories to determine the Riemannian mean after the third trial. $\bar{\text{P}}$ was iterated using the following equation as a new SPD matrix was added to the manifold:

(4) $${\bar{\text{P}}}_{t+1}={\left({\bar{\text{P}}}_{t}\right)}^{1/2}\,{\left[{\left({\bar{\text{P}}}_{t}\right)}^{-1/2}\,\text{P}\,{\left({\bar{\text{P}}}_{t}\right)}^{-1/2}\right]}^{1/\alpha }\,{\left({\bar{\text{P}}}_{t}\right)}^{1/2}$$

where the matrix **P** denotes the new matrix, ${\bar{\text{P}}}_{t}$ is the Riemannian mean matrix from the previous iteration, and α represents the speed of the adaptation. Besides, the mean absolute deviation of the set of **P**_*i*_ is the average distance between each **P**_*i*_ and the Riemannian mean $\bar{\text{P}}$:

(5) $$\sigma_{P}=\frac{1}{N}\,\sum_{i=1}^{N}\delta_{R}\,\left({\text{P}}_{i},\bar{\text{P}}\right)$$

#### Artifact Detection

We used a multivariate EEG artifact detection method called Riemannian Potato, which estimates a reference point in Riemannian manifold and rejects any EEG segment that has a long Riemannian distance from the reference point (Barachant et al., 2013a). The EEGs were band-pass filtered between 1 and 30 Hz before calculating the covariance matrix. The initialization of the reference point was the Riemannian mean of 10 1-s EEG covariance matrices of the 10-s staying-still-period EEG measurement recorded before the first run. The reference point was adapted during the whole session using Eq. (4) with α of 100. The reference point was only adjusted when the current EEG segment was of good quality.

The initial threshold *th* was defined by the mean μ and the standard deviation σ among the Riemannian distance between the 10 1-s EEG covariance matrices of 10-s staying-still-period and the initial reference point, that is:

(6) t⁢h=μ+2.5⁢σ

When the Riemannian distance between the current EEG and the reference point is greater than *th*, the current EEG segment is considered as an artifact. μ and σ are also iterated mildly by every clean EEG.

(7) $$\begin{array}{c} \mu_{t+1}=\left(1-w\right)*\mu_{t}+w*\delta_{t+1},\\ \sigma_{t+1}=\sqrt{\left(1-w\right)*\sigma_{t}^{2}+w*{\left(\delta_{t+1}-\mu_{t+1}\right)}^{2}} \end{array}$$

where δ_*t+1*_ represents the Riemannian distance between the newly added clean EEG and the reference point, which is calculated using Eq. (2). μ_*t*_ and σ_*t*_ represent the mean and standard deviation in the previous iteration. *w* represents the speed of the adaptation, which equals 0.01. This method revealed gnashing, sleepiness, head movements, and jaw movements expressed during the experiment.

In addition, another artifact removal was performed using ICA (independent component analysis), resulting in loss of about 20% of the trial, before calculating the sensorimotor rhythm (SMR) discriminancy, since we tended to use purer EEG signal when analyzing the SMR discriminancy. Specifically, after removing the artifacts using Riemannian artifact detection method, the EEG data were decomposed by ICA. Then, we rejected the ICs (independent components) by scalp maps and rejected the epochs of IC time courses according to the abnormal values, abnormal trends, abnormal distributions, improbable data, and abnormal spectra.

#### Diffusion Map

The Riemannian distance between each pair of EEG covariances is an *N* × *N* symmetric matrix. We introduced a manifold learning method, diffusion map, to dimensionally reduce the Riemannian distance matrix into two dimensions. A diffusion map computes a family of embeddings of a dataset into a low-dimensional Euclidean space whose coordinates can be calculated from the eigenvectors and eigenvalues of a diffusion operator on the data (Coifman et al., 2005; Krauss et al., 2018). Four steps are included in this process. First, a Gaussian kernel matrix **K** is constructed:

(8) $${\text{K}}_{i\,j}=\exp\left(-\frac{\delta^{2}\,\left({\text{P}}_{i},{\text{P}}_{j}\right)}{\varepsilon }\right)$$

where ε is the kernel scale, which was equal to 20 in our case. Next, the diffusion matrix is created by normalizing the rows of the kernel matrix:

(9) $${\text{K}}^{\text{norm}}={\text{D}}^{-1/2}\,{\text{KD}}^{-1/2}$$

Where **K**^norm^ represents the diffusion matrix and **D** is a diagonal matrix whose elements are the sums of rows of **K**. The eigenvectors of **K**^norm^ are calculated, φ_*i*_ ∈ *ℝ*^*N*^, and the eigenvalues are λ_0_,λ_1_,⋯,λ_*N*−1_. Finally, the elements in 𝒫(*n*) are mapped to a *d*-dimensional Euclidean space using the *d* dominant eigenvectors and eigenvalues:

(10) $${\text{Y}}_{i}^{\prime }=\left[\begin{array}{c} \lambda_{1}\,\varphi_{1}^{i}\\ \lambda_{2}\,\varphi_{2}^{i}\\ \vdots \\ \lambda_{d}\,\varphi_{d}^{i} \end{array}\right]$$

where *d* was set to 2 here; **Y**_*i*_ denotes the *x*- and *y*-coordinates for *i*th element on the feedback screen. The distance between points in Euclidean space is equal to the “diffusion distance” between probability distributions centered at those points.

### Evaluation of Subjects’ Performance

Three metrics were used to evaluate the subjects’ learning effects: class distinctiveness, discriminancy of EEG features, and classification accuracy.

#### Class Distinctiveness

The distinctiveness of two patterns can be expressed by the Fisher criterion, where the distance and absolute deviation can be replaced by properties of Riemannian geometry (Lotte and Jeunet, 2018). We used the metric *ClassDis* to determine the distinctiveness of EEG pattern pairs (class *A* and class *B*):

(11) $$C\,l\,a\,s\,s\,D\,i\,s\,\left(A,B\right)=\frac{\delta_{R}\,\left({\bar{\text{P}}}_{A},{\bar{\text{P}}}_{B}\right)}{1/2\,\left(\sigma_{P_{A}}+\sigma_{P_{B}}\right)}$$

where ${\bar{\text{P}}}_{A}$, ${\bar{\text{P}}}_{B}$, and σ_*P_A_*_, σ_*P_B_*_ are the Riemannian mean and the mean absolute deviation of EEG covariance matrices of class *A* and class *B*, respectively. The Riemannian distance δ_*R*_() and mean absolute deviation σ_*c*_ were computed by Eqs (2, 5), respectively. A higher *ClassDis* reflects smaller class dispersion and greater distance between classes.

#### EEG Feature Discriminancy

In addition to measuring the learning effects with class distinctiveness, we also assessed the discriminancy of EEG features (Perdikis et al., 2018). The discriminancy of a given spatio-spectral EEG feature, which corresponds to a specific EEG channel and a frequency band, for two MI tasks was quantified by the Fisher score:

(12) $$F\,S=\frac{\left|\mu_{1}-\mu_{2}\right|}{\sqrt{s_{1}^{2}+s_{2}^{2}}}$$

where μ_*1*_ and μ_*2*_ denote the means of the feature’s sample values for left hand-MI and right hand-MI, and *s*_*1*_, *s*_*2*_ denote the standard deviations. The EEG features were first spatially filtered with a Laplacian derivation, and then the power spectral density of each channel was computed with 2-Hz resolution in 1-s windows sliding every 62.5 ms. The discriminancy over 30 channels physiologically relevant to MI topographic and the specific spectral band was computed as the average Fisher score of all features. In addition, the ICA artifact removal method was performed after Riemannian Potato method when calculating the SMR discriminancy.

#### Classification Accuracy

Rather than only the *ClassDis* and *FS* metrics, we also calculated the off-line classification accuracy (CA) to investigate the performance of subjects across the 3-day-training process. EEG signals varied at the run scale, so we employed fivefold cross-validation on 160 EEG samples within each run, where a 4-s EEG measurement was divided into four 1-s EEG segments in each trial. We used a supervised Riemannian classifier called minimum distance to Riemannian mean to obtain off-line CA (Barachant et al., 2012). It computed the Riemannian distance between the unlabeled EEG covariance matrix and the Riemannian Mean of every intra-class covariance matrix. The class with the smallest Riemannian distance corresponds to the one of the unlabeled EEG.

### Statistical Analysis

We conducted planned comparisons on the first and last session as well as the first and last day for all cases. The first and last session (day) of the averaged learning curve among subjects were compared and tested for significant differences by using the polynomial contrasts of one-way repeated-measures ANOVA model. Mauchly’s test was used to check for sphericity in the ANOVA. Greenhouse–Geisser epsilon values were used to account for any violations of sphericity. The first and last session (day) of the learning curve per subject were compared and tested for significant differences at the 95% confidence interval using paired, two-sided Wilcoxon rank-sum tests. The training effects are reported here as Pearson correlation coefficients with significance at the 95% confidence interval through Student’s *t*-test distribution. The relationship among the evaluation metrics *ClassDis*, *FS*, and CA, was also determined by the Pearson correlation.

### Advanced Training Protocol

Researchers have suggested that subjects can only increase their MI-BCI performance in a more specific frequency band even when using a broad EEG frequency band in training feedback (Lotte and Jeunet, 2018). Thus, restraining the feedback tasks to focus on an optimal frequency band may increase the efficiency of learning for each subject. We used the 8- to 30-Hz frequency band in the first two sessions of our experiment and a subject-specific 4-Hz wide frequency band in the remaining four sessions. The broad frequency band (8–30 Hz) was decomposed into a total of 19 frequency bands, namely, 8–12, 9–13, …, 26–30 Hz, i.e., every 4-Hz wide band with a 1-Hz step between consecutive bands. The *ClassDis* was calculated for these 19 bands using Eq. (11): *ClassDis*_*f*_,*f* = 1,…,19. The *ClassDis*_*f*_ was then averaged among the 10 runs in the first two sessions, and the frequency band corresponding to the highest *ClassDis*_*f*_ was chosen as the optimal EEG band for the remaining four sessions.

Brain–computer interface learning should necessarily take place in variables fed through given feedback. These variables differed between the first day and the next 2 days of our training process. Thus, the learning curve of *ClassDis* and EEG feature discriminancy was established based on three conditions. The first was the “actual, online” result, which reflects what the subjects were seeing from the visual feedback. The EEG covariance matrix was computed on the broadband (8–30 Hz) for sessions 1 and 2 and on the subject-specific optimal bands for sessions 3–6. Second was the “broadband” result, in which EEG covariance matrix was calculated on the broadbands all over. Third was the “subject-specific” result, in which the EEG covariance matrix was computed only on the subject-specific bands throughout.

## Results

We first determined the class distinctiveness and neurophysiological evidence of subject learning. Next, we determined the optimal frequency band across sessions and the influence of the different frequency bands on the location of feedback points. Finally, we tested the off-line classification accuracies throughout the training process.

### Class Distinctiveness Results

**FIGURE 3:**
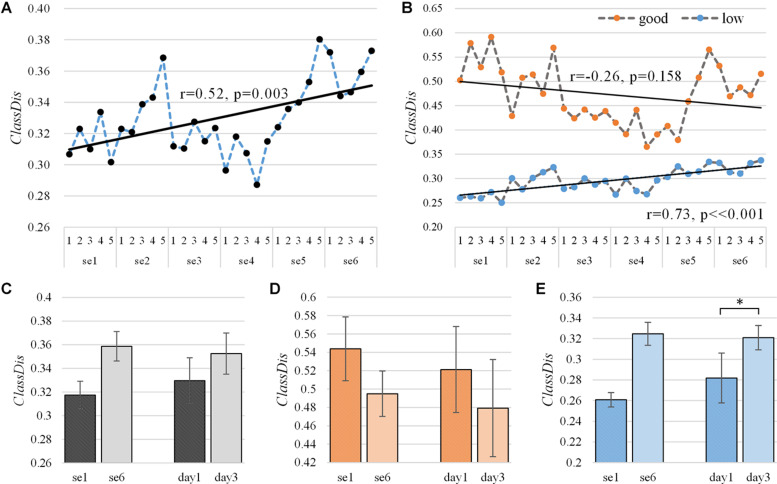
Average “actual, online” learning curve of *ClassDis* for two MI tasks across six sessions **(A)** among 10 subjects and **(B)** among subjects with good (orange) and low (blue) MI-BCI skill. The corresponding linear fits and Pearson correlation coefficients demonstrate training effects. Average and standard deviation of *ClassDis* in first and last session (day), **(C)** among 10 subjects, **(D)** among subjects with good MI-BCI skill, and **(E)** among subjects with low MI-BCI skill. The first and last session (day) of *ClassDis* tested for significant differences using the polynomial contrasts of one-way repeated-measures ANOVA model, *p < 0.05. The dependent variable and the independent variable (within-subject factor) are *ClassDis* and run, respectively, and *ClassDis* ranges from 0 to 1.

We employed the metric *ClassDis* to measure the distinctiveness between left hand-MI and right hand-MI EEG signals throughout the training process. The learning curve of *ClassDis* is provided in three versions: “actual, online,” “broadband,” and “subject-specific.” The average “actual, online” *ClassDis* of 10 subjects throughout training is shown in Figure 3A. The blue dashed line represents the learning curve for 30 runs and the black line represents the corresponding linear fit. Significant positive Pearson correlations between *ClassDis* and the run index indicated the existence of a significant training effect on class distinctiveness (*r* = 0.52, *p* = 0.003, and *N* = 30). Figure 3C shows where our training procedure increased the *ClassDis* from 0.315 ± 0.012 (*N* = 5, first session) to 0.359 ± 0.012 (*N* = 5, last session) and similarly from 0.327 ± 0.019 (*N* = 10, first day) to 0.353 ± 0.017 (*N* = 10, last day). These improvements did not show any significance according to the polynomial contrasts of one-way repeated-measures ANOVA model though [session 1 vs. session 6: *F*(1,9) = 0.984, *p* = 0.347, and partial η^2^ = 0.203; day 1 vs. day 3: *F*(1,9) = 1.172, *p* = 0.307, and partial η^2^ = 0.115].

Next, we divided the subjects with good MI-BCI skill and low MI-BCI skill into separate categories. According to the *ClassDis* value of the first session, subjects with *ClassDis* greater than 0.317 (equal to the average *ClassDis* among 10 subjects in session 1) are good-performance subjects, whereas those with *ClassDis* less than 0.317 are low-performance subjects. As shown in Table 1 (Column 4), s1, s7, and, s9 are good-performance subjects and the others are low-performance subjects. It appears that all three good-performance subjects have strong right-handedness.

The average “actual, online” *ClassDis* of three good-performance subjects and seven low-performance subjects across sessions are shown in Figure 3B; there is a slightly decreasing trend of the average *ClassDis* for good-performance subjects (orange dots) over the runs (*r* = −0.26, *p* = 0.158, and *N* = 30). On the contrary, Figure 3B shows a statistically significant increasing trend in the average *ClassDis* for low-performance subjects (blue dots) over the runs (*r* = 0.73, *p* < 0.001, and *N* = 30). The *ClassDis* for good-performance subjects decreased from 0.544 ± 0.035 (*N* = 5, first session) to 0.495 ± 0.025 (*N* = 5, last session) and similarly from 0.521 ± 0.047 (*N* = 10, first day) to 0.479 ± 0.053 (*N* = 10, last day), as shown in Figure 3D, both with no significant difference [session 1 vs. session 6: *F*(1,2) = 0.336, *p* = 0.621, and partial η^2^ = 0.144; day1 vs. day3: *F*(1,2) = 2.013, *p* = 0.292, and partial η^2^ = 0.502]. Figure 3E shows where our training procedure increased the *ClassDis* for low-performance subject from 0.261 ± 0.007 (*N* = 5, first session) to 0.325 ± 0.011 (*N* = 5, last session) and similarly from 0.282 ± 0.024 (*N* = 10, first day) to 0.321 ± 0.012 (*N* = 10, last day). The improvement from the first day to the last day showed a statistical significance [session 1 vs. session 6: *F*(1,6) = 3.423, *p* = 0.114, and partial η^2^ = 0.363; day1 vs. day3: *F*(1,6) = 6.397, *p* = 0.045, and partial η^2^ = 0.516].

The learning metric *ClassDis* per subject is shown in Supplementary Figure 1. The overall learning effects, which despite clear trends, may be biased by certain subjects. We found that 30% of the subjects show a statistically significant increasing trend of the “online, actual” *ClassDis* over the runs (s3: *r* = 0.40, *p* = 0.027; s5: *r* = 0.54, *p* = 0.002; and s6: *r* = 0.42, *p* = 0.021, and *N* = 30); however, 20% of them show a decreasing trend (s1: *r* = −0.07, *p* = 0.724; s7: *r* = −0.54, *p* = 0.002, and *N* = 30). The subjects who reveal a statistically significant increasing trend in terms of the learning metric were considered to be “learners,” and those who show a decreasing trend over the proposed training procedure were considered “non-learners”; all learners were also low-performance subjects.

The average *ClassDis* value of 10 subjects throughout training in terms of “broadband” and “subject-specific” versions are shown in Supplementary Figure 2. Significant positive Pearson correlations between the learning metric value and run index confirm a significant training effect both on “broadband” *ClassDis* (*r* = 0.81, *p* < 0.001, and *N* = 30) and “subject-specific” *ClassDis* (*r* = 0.80, *p* < 0.001, and *N* = 30). The proposed training procedure increased the *ClassDis* in “subject-specific” version from 0.330 ± 0.019 (*N* = 10, first day) to 0.390 ± 0.025 (*N* = 10, last day), and the improvement is statistically significant [*F*(1,9) = 5.568, *p* = 0.043, and partial η^2^ = 0.382]. The *ClassDis* for each subject in these two versions were provided in Supplementary Figures 3, 4. On the “broadband” *ClassDis*, 40% of the subjects are learners (s1: *r* = 0.36, *p* = 0.048; s3: *r* = 0.60, *p* < 0.001; s5: *r* = 0.54, *p* = 0.002; and s6: *r* = 0.55, *p* = 0.002) and 20% are non-learners (s7: *r* = −0.31, *p* = 0.099; s8: *r* = −0.05, *p* = 0.788). On the “subject-specific” *ClassDis*, 30% of the subjects are learners (s1: *r* = 0.44, *p* = 0.016; s3: *r* = 0.59, *p* < 0.001; and s5: *r* = 0.53, *p* = 0.002) and 20% are non-learners (s7: *r* = −0.26, *p* = 0.170; s8: *r* = −0.07, *p* = 0.714).

### Neurophysiological Evidence of Subject Learning

**FIGURE 4:**
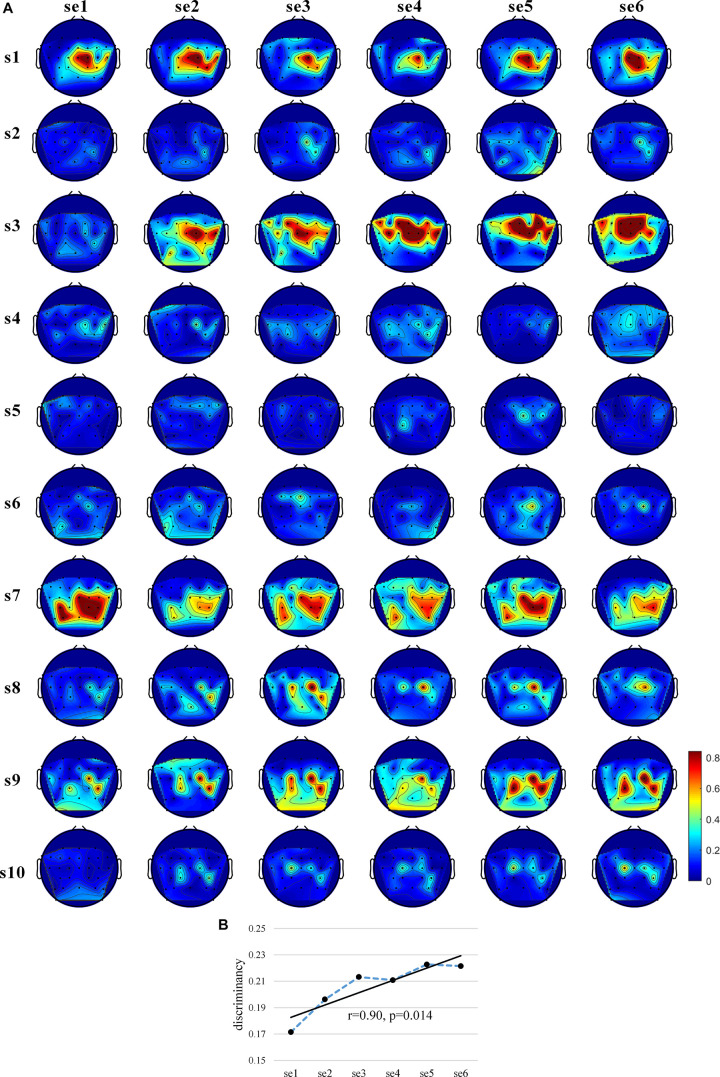
Discriminancy of EEG features. **(A)** The “actual, online” (8–30 Hz) topographic maps of discriminancy per training session on 30 EEG channel locations over sensorimotor cortex. Red indicates high discriminancy between left hand-MI and right hand-MI tasks employed by 10 subjects. Discriminancy of each channel is quantified as Fisher score of power spectral density distributions of EEGs for two MI tasks in “actual, online” frequency band within each session. **(B)** Average “actual, online” learning curve of EEG feature discriminancy for 10 subjects. Discriminancy value of each session calculated by averaging Fisher scores among all 30 channels. Corresponding linear fits and Pearson correlation coefficients are reported to indicate training effects.

In order to assess the existence of subject learning effects directly at the EEG feature level, we used the Fisher score metrics to measure the separability of the EEG spectral distributions for the two MI tasks. Figure 4A shows the “actual, online” topographic maps of discriminancy. s1, s7, and s9 largely maintained the same brain pattern (SMR discriminancy in channel C2 for s1 and s3, in channel C2, CP4, and Cp1 for s9); s2 showed an increasingly obvious SMR discriminancy in channel C2, even increasing the strength of medial modulation (channel C1, Cz, FC1, FCz, and Fc2) in the last session. On the contrary, s2, s4, s5, s6, and s10 showed subtle and unstable SMR discriminancy throughout the training process, though some showed a slight increase.

Figure 4B shows the average “actual, online” learning curve of the discriminancy for 10 subjects across all sessions. The significant positive Pearson correlations between the SMR discriminancy and session index prove the existence of a significant training effects in terms of the neurophysiological evidence (*r* = 0.90, *p* = 0.014, and *N* = 6). Supplementary Figure 5 shows the “actual, online” learning curve of the discriminancy per subject. The proposed feedback protocol appears to have been effective in inducing an emerging SMR pattern (“actual, online” frequency band, over all 30 channels) for some of the subjects. More specifically, it substantiated a significant enhancement over the sessions for s3, s9, and s10 (s3: *r* = 0.81, *p* = 0.042; s9: *r* = 0.88, *p* = 0.022; and s10: *r* = 0.95, *p* = 0.004). Supplementary Figures 6–9 show the discriminancy maps and corresponding learning curves for “broadband” and “subject-specific” frequency bands. Moreover, we calculated the average “actual, online” discriminancy of subjects’ SMRs among 10 channels that the proposed feedback employed in order to estimate the relationship between EEG discriminancy and *ClassDis* (calculated using only 10 channels). It indicates that FS correlated closely with the metric “actual, online” *ClassDis* (*N* = 60, *r* = 0.83, and *p* < 0.001). Hence, the increased SMR modulation seems be crucial for enhanced MI-BCI skill.

### The Optimal Frequency Band Results

The optimal frequency band for each subject played an important role in MI-BCI training, as the effect of training in the last 2 days was dependent on the selected frequency band. The optimal EEG frequency bands for the first two sessions and all six sessions are shown in Table 1 (Columns 5 and 6). We found that the optimal frequency bands for the first two sessions are consistent with those for all sessions, notably the same as the best or the second-best frequency band for all sessions. The subjects were assessed according to the average *ClassDis* of the six sessions. The average *ClassDis*_*f*_,*f* = 1,…,19 of good and low-performance subjects are shown in Supplementary Figures 10A,B, respectively. The curves of *ClassDis*_*f*_ for 30 runs (fine colorful lines) in Supplementary Figure 10A are in close accordance with the averaged curve among them (thick black line). The thick black line reaches its highest point at 10–14 Hz, then declines, and then increases again before leveling off in the beta band. As shown in Supplementary Figure 10B, the 30 curves of *ClassDis*_*f*_ are also consistent with the averaged curve among them but do not change much with the frequency band. The highest *ClassDis*_*f*_ is 11–15 Hz and the second highest is 24–28 Hz, belonging to alpha and beta bands, respectively.

**FIGURE 5:**
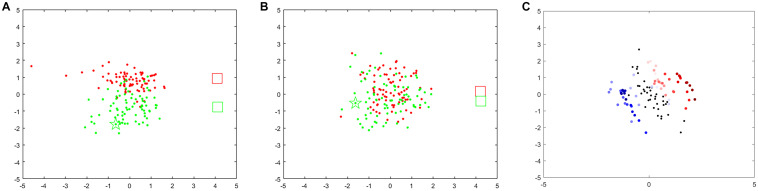
Feedback points for different frequency bands and time windows for s1. When training-run frequency band is 9–13 Hz **(A)** or 13–17 Hz **(B)**. Red points denote 1-s feedback, red rectangle denotes Riemannian mean of feedback for left hand-MI, green points and green rectangle denote feedback and Riemannian mean for right hand-MI. **(C)** Feedback points for two trials (red marks left hand-MI and blue marks right hand-MI). Color changes from transparent to dark represents time from appearance of cue to end of MI task within one trial. Black point denotes resting state (1 s before cue).

We also investigated the influence of the different frequency bands and time windows on the feedback point diagram. As shown in Figure 5A, point clusters for left hand-MI (red) and right hand-MI (green) can be clearly distinguished when the optimal frequency band is chosen. Their corresponding Riemannian mean (two rectangles) are also far apart. As compared to Figure 5A, red and green point clusters in Figure 5B show significant overlapping, and the two rectangles are very close to each other.

Figure 5C shows variations in performance over time expressed as a feedback point diagram for two trials within a run (red for left hand-MI and blue for right hand-MI). Two EEG trials belonging to two MI tasks are shown as an example. A 6-s EEG segment from the beginning of a trial to the end of the imagination task was decomposed into 51 time windows using a 1-s time window with a 0.1-s step. The first 11 time windows belong to the resting state, and the last 40 belong to the MI tasks. Each black, red, and blue point was computed within the corresponding time window separately. Most of the transparent red and blue points fall around the black points; the red and blue points fall farther from the black points as the color deepens. The dark points belonging to each class accumulate near the two sides of the black points. This suggests that the brain needs time to transition from the resting state to a stable SMR. The distinctiveness between two MI tasks reaches its maximum at an identifiable time point.

### CA Results

**FIGURE 6:**
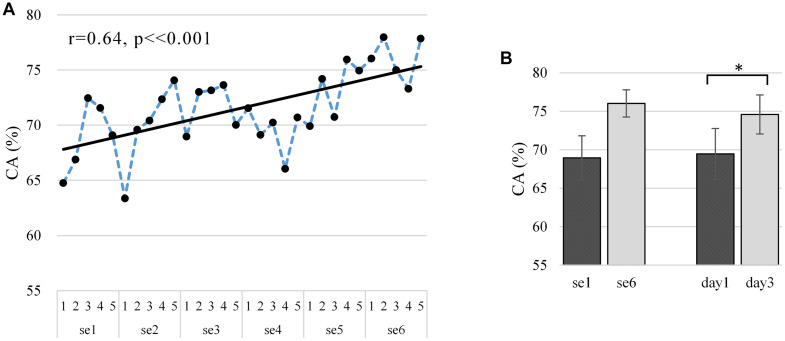
**(A)** Average “broadband” learning curve of CA for two MI tasks across six sessions among 10 subjects. The corresponding linear fits and Pearson correlation coefficients demonstrate training effects. **(B)** Average and standard deviation of CA in first and last session (day) among 10 subjects. The first and last session (day) of CA tested for significant differences using the polynomial contrasts of one-way repeated-measures ANOVA model, *p < 0.05. The dependent variable and the independent variable (within-subject factor) are CA and run, respectively, and CA is ranging from 0 to 100%.

Figure 6A shows the off-line CA at the run scale in the “broadband” version. CA varied largely between subjects (mean 0.716 ± 0.136), ranging from 0.560 to 0.916 (well-controlled). The Pearson correlations between the CA and run index are statistically significantly positive (*r* = 0.64, *p* < 0.001, and *N* = 30), which proves the existence of a significant training effect on CA. As shown in Figure 6B, our training procedure increased CA from 0.689 ± 0.029 (*N* = 5, first session) to 0.760 ± 0.018 (*N* = 5, last session) and similarly from 0.694 ± 0.033 (*N* = 10, first day) to 0.746 ± 0.025 (*N* = 10, last day). The improvement from the first day to the last day showed a statistical significance as per the polynomial contrasts of one-way repeated-measures ANOVA model [session 1 vs. session 6: *F*(1,9) = 4.294, *p* = 0.068, and partial η^2^ = 0.323; day 1 vs. day 3: *F*(1,9) = 8.049, *p* = 0.019, and partial η^2^ = 0.472]. The learning metric CA per subject is given in Supplementary Figure 11, where 30% of the subjects show a statistically significant increasing trend in the “broadband” CA over the runs (s3: *r* = 0.64, *p* < 0.001; s8: *r* = 0.41, *p* = 0.026; and s9: *r* = 0.47, *p* = 0.009), though 20% of the subjects show a decreasing trend (s2: *r* = -0.03, *p* = 0.880; s7: *r* = -0.34, *p* = 0.063). The learning curves in Figure 6A and Supplementary Figure 2A show similar upward trends, with a statistically significant Pearson correlation (*r* = 0.773, *p* < 0.001).

## Discussion

The proposed approach takes advantage of the Riemannian geometry method and, instead of learning a hyperplane, iteratively calculates the Riemannian distance among covariance matrices that are translated into the feedback visualization through a diffusion map process. This approach serves the purpose of, on the one hand, enabling a compressed visualization of the overall brain pattern, which can be straightforwardly processed by the subject and, still, depends on all features without discarding anything. On the other hand, the Riemannian geometry-based feedback approach aims to visualize the same signal that will eventually control the final actuator in the BCI system, which greatly reduces the adaptation time from BCI training to the application (Perdikis and Millan, 2020). Besides, it suggested that only smoothly adapting feedback is preferred, which enhances the subject’s learning capacities. In fact, the feedback points as well as the Riemannian mean points in the proposed training procedure move slightly, namely, the position of each feedback is fixed in a small area, which satisfies the requirements for feedback adaptation.

The main contribution of this work is the provision of quantitative evidence regarding the proposed online data visualization feedback protocol. The linear fit of the average learning curve of “actual, online” *ClassDis* moved upward, which indicated the existence of the overall training effect. Compared with the first day, *ClassDis* values under the subject-specific frequency band were significantly higher on the third day. However, only 3 out of 10 of the subjects were learners, and all were low-performance subjects. Thus, training effects in terms of *ClassDis* tended to be larger in low performers, since they were not affected by ceiling effects, unlike good performers. The result is in accordance with observations made by Meng and He (2019), who found a significant improvement in ITR only in the low-performance group.

Besides, the average *ClassDis* decreased in session 4 among 6 out of 10 subjects compared to session 3. This drop may be attributable to the advanced training protocol. We replaced the training feedback with the advanced protocol from the second day of training onward, where a narrower frequency band was used when estimating SPDs. SPDs with a narrower frequency band contained less distinctiveness information for the two MI tasks, making it harder for subjects to separate point clusters from the two classes. We infer that the difficulty led to some maladjustment in the subjects, therefore decreasing the *ClassDis* values. However, the averaged *ClassDis* for both good- and low-performance subjects increased steadily over the last three sessions. To this effect, the advanced training protocol had a positive effect on subjects’ MI-BCI performance in the long term.

We also analyzed the neurophysiological evidence of subject learning using the SMR discriminancy. The intensity of the average SMR discriminancy over the sensorimotor area varied largely among the subjects. The training procedure effectively induced an emerging SMR pattern for only three of the subjects. Three learners under these two metrics diverged. The reason is that, on the one hand, the slight difference was insufficient to separate two tasks despite the upward trend in SMR discriminancy. On the other hand, the metric of EEG feature discriminancy represented the pure SMR due to the additional artifacts removal method using ICA. Conversely, the distinctiveness between two classes may be enhanced by non-SMR features. Besides, according to the upward trend of s2, s4, s8, and s10, we may speculate that studies, possibly with more training sessions, are required to enhance the training effect.

Thus, from the results of the class distinctiveness and the discriminancy of EEG feature, we concluded that besides some slight trends, no sold, reliable evidence of learning has been found, and that future work should ultimately answer this question with more extensive and better-designed studies.

We found that the optimal frequency bands for good-performance subjects were highly consistent among the 30 runs and all belong to the alpha band (10–14 Hz). The optimal frequency bands for low-performance subjects were also consistent among 30 runs and belong to both alpha and beta bands. This finding may be useful for the selection of optimal frequency bands in MI-BCI application. The frequency band corresponding to the highest averaged *ClassDis*_*f*_ could be selected for calibration sessions and treated as the permanent optimal frequency band for other MI-BCI activities. The off-line CA results showed a significantly positive Pearson correlations between the “broadband” CA and the run index. The CA variations were also closely correlated with the *ClassDis* variations. The Riemannian distance properties of EEGs within a given frequency band were taken into account in both metrics, which may account for this.

The Riemannian Mean of two categories (two rectangles on the feedback screen) was shown in order to guarantee a relatively small change, visually, from one feedback to the next in every second. Indeed, the projection variability was high initially. After few trials, each feedback point moved slightly. The position of each feedback was fixed in a small area, which did not affect the visualization as perceived by the subjects. Unexpectedly, however, the projection map may have flipped symmetrically along the *x*-axis or *y*-axis. Mathematically, the sign of the coordinate of the feedback point changed as the experiment progressed. To mitigate the flipped projections, we added a projection of the Riemannian mean for two categories represented as two rectangles on the feedback screen. The feedback points of one category could be fixed by making the coordinate sign of the rectangle of this category invariant, since the rectangle of one category was always on the same side as the feedback points of that category.

The one-by-one appearance of feedback points by diffusion map projection after fixing the coordinate sign of the Riemannian mean is shown in Supplementary Videos 2, 3. This appeared to be an effective method to prevent projection variability. Unfortunately, this did not resolve the excessive projection variability when only a few points were displayed. Thus, the feedback points initially had a negligible instructional effect on subjects. This problem will need to be remedied in a future study. Additionally, as the artifacts were far away from the normal EEG measurements in terms of Riemannian distance, their presence markedly affected the 2D projection representation; the normal EEG feedback points clustered in a line on the 2D screen. Thus, the artifact removal made a significant contribution for preventing the position changes.

We chose five pairs of EEG electrodes on the bilateral sensorimotor cortex. Without any subject-specific channel selection, we investigated the power distribution throughout the sensorimotor cortex instead of the power of one or a few fixed electrodes. Ten electrodes were appropriate; any additional electrodes were more likely to introduce noise when estimating the covariance matrix. However, the estimation precision of the 10 × 10 covariance matrix from 1-s EEG data was relatively unclear.

A new point was presented on the feedback screen every 1 s in accordance with our protocol. The CPU of our computer is Intel Core i7 with 32-G RAM. The computational demand of a new projection for each new EEG covariance matrix was approximately 0.43 s (in real time). The processing time increased with the number of EEG covariance matrices during one run, as a new EEG covariance matrix required the computation of Riemannian distances from all the previous covariance matrices. Implementing a faster-updated rate was difficult for the proposed feedback protocol, as a faster pace would have necessitated more feedback points, to the point that the processing time may have exceeded the update time in the later stage of a run, thus causing the experiment to collapse.

We carefully considered the design of our training experiment. Before the experiment started, we only prompted subjects to perform kinesthetic—rather than visual—MI tasks but did not prescribe a specific mental strategy. Therefore, the subjects could fully explore the mental strategies with the most remarkable difference between two hands-MI according to the real-time feedback points.

There are two limitations in our experimental design that will need to be resolved in any follow-up work. First, we included subjects with previous BCI experience, which confounded the training effects. When investigating whether subjects can learn a skill, it is necessary to ensure that no subject is already an expert or has even been exposed to the training previously. Second, we intended to prove that the proposed approach is an alternative to the conventional cursor feedback training regarding subject learning; unfortunately, we designed an uncontrolled experiment and thus could not fully determine whether the proposed approach is better than the conventional one. Two pieces of literature with relatively long experiment length conducted uncontrolled experiment (Perdikis et al., 2018; Meng and He, 2019). Barsotti et al. (2018) and Penaloza et al. (2018) obtained significant differences between the proposed feedback method and the conventional method; however, the experiment length all lasted for only 1 day. In our opinion, the 1-day experimental design suffered from a lack of longitudinal evaluation (Perdikis and Millan, 2020). Hence, even if it could be observed a significant difference, it cannot be concluded whether a new feedback method has contributed, or the observed effects regard short-term adaptation of the subjects to the new interface. In addition, Sollfrank et al. (2016) provided a well-designed controlled experiment; however, no significant difference was found between the controlled feedback and the multi-modal funnel feedback. We will continue completing the controlled experiment for further research referring to this experimental paradigm.

The proposed feedback protocol focused on subject learning. We did not explore machine learning methods in this study. The compression points of 160 EEG measurements in one run (when no artifact was detected) appeared in turn. By the next run, all the previous 160 points were cleared from the screen. This may have left subjects unable to continuously learn the prior training experiments. However, it was not feasible to simply display the previous compression points on the screen, due to the intrinsic non-stationarity of EEG data and the probability of converting different mental strategies for the subject. Perdikis and Millan (2020) concluded that subject learning capacities can be fostered as if machine learning adaptation is only enabled at the beginning of new sessions until non-stationarity effects are alleviated. In the future, we will employ the affine transformation method at the beginning of new sessions to allow for continuous learning (Zanini et al., 2018).

## Conclusion

In this study, we established a Riemannian geometry-based data visualization MI-BCI training feedback protocol that allows subjects to learn to modulate their SMRs by separating points of different colors and then centralizing points of the same color on a feedback screen. The proposed protocol does not require training calibration and provides continuous feedback over a long period of time. We observed overall training effects among 10 subjects after 3-day training. However, no solid, reliable evidence of learning was found in most of the subjects. We speculate that the subjects’ training efficacy could be improved by longitudinal experimentation according to the neurophysiological evidence. We found increases in *ClassDis* for the last three sessions, which suggests that the proposed protocol has positive effects in the long term. In the future, our observations can be strengthened by further improving the protocol design and the experimental design.

## Data Availability Statement

The raw data supporting the conclusions of this article will be made available by the authors, without undue reservation.

## Ethics Statement

The studies involving human participants were reviewed and approved by Northwestern Polytechnical University Medical and Experimental Animal Ethics Committee. The patients/participants provided their written informed consent to participate in this study.

## Author Contributions

XD and SYX conceptualized the idea and proposed the experimental design. XD programmed and performed all data analysis and as well as wrote the manuscript at all stages. SYX and XX revised the manuscript at all stages. KO revised the manuscript. XD, YC, and ZW conducted the experiments. All authors read and approved the final manuscript.

## Conflict of Interest

The authors declare that the research was conducted in the absence of any commercial or financial relationships that could be construed as a potential conflict of interest.
