# Supplementary material for: An Online Data Visualization Feedback Protocol for Motor Imagery-Based BCI Training
Source: Front Hum Neurosci. 2021 Jun 7;15:625983. doi: 10.3389/fnhum.2021.625983 (PMC8215169; doi:10.3389/fnhum.2021.625983)
Supplement: Supplementary file 1 [file Table_1.docx]

Supplementary Material

# Supplementary Figures and Videos

## Supplementary Figures


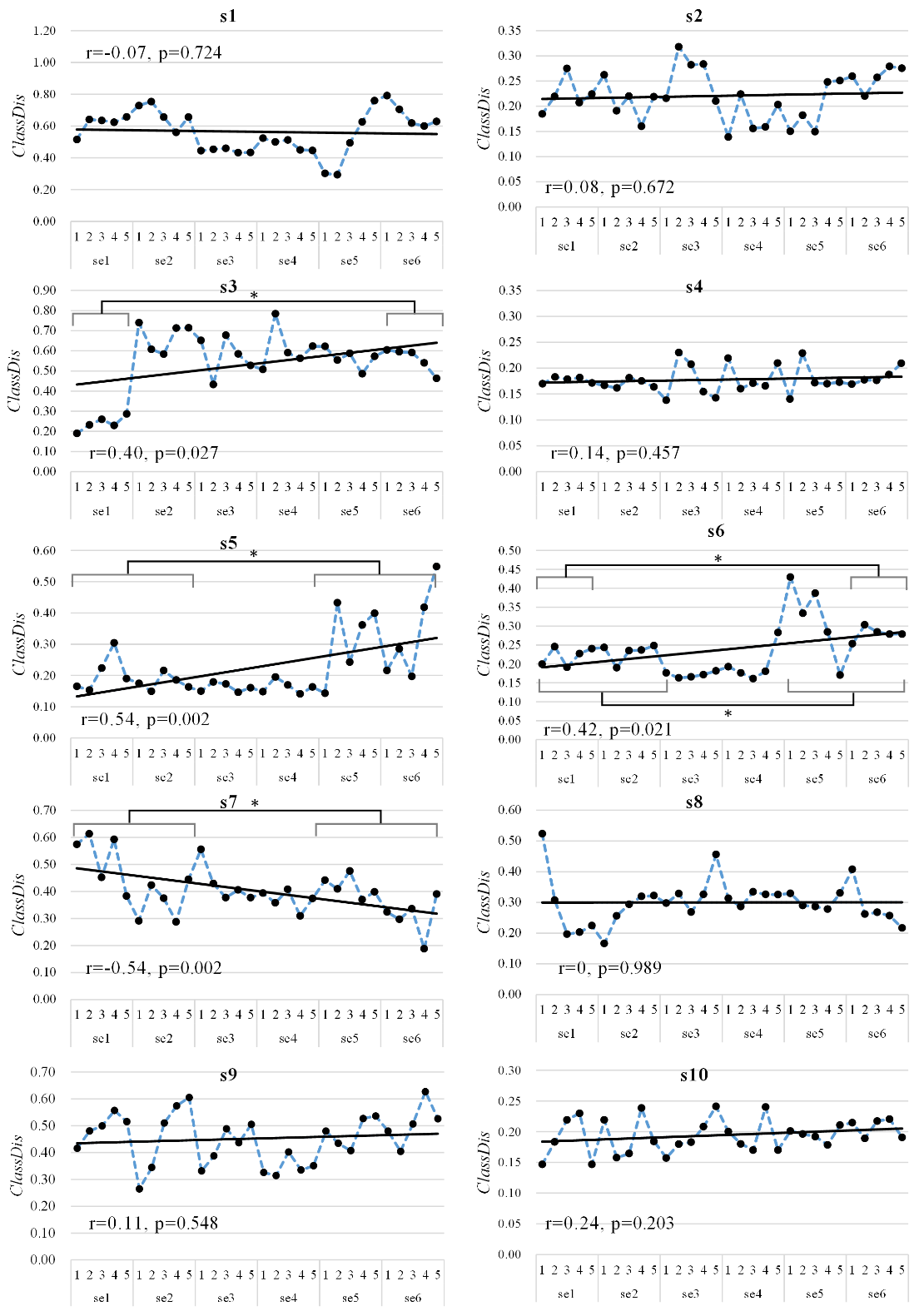


**Supplementary Figure 1.** The “actual, online” learning curve of *ClassDis* for two MI tasks throughout training for each subject. The corresponding linear fits and Pearson correlation coefficients demonstrate training effects. The first and last session (day) of *ClassDis* were tested for significant differences using two-sided Wilcoxon ranksum tests, $*p<0.05$.


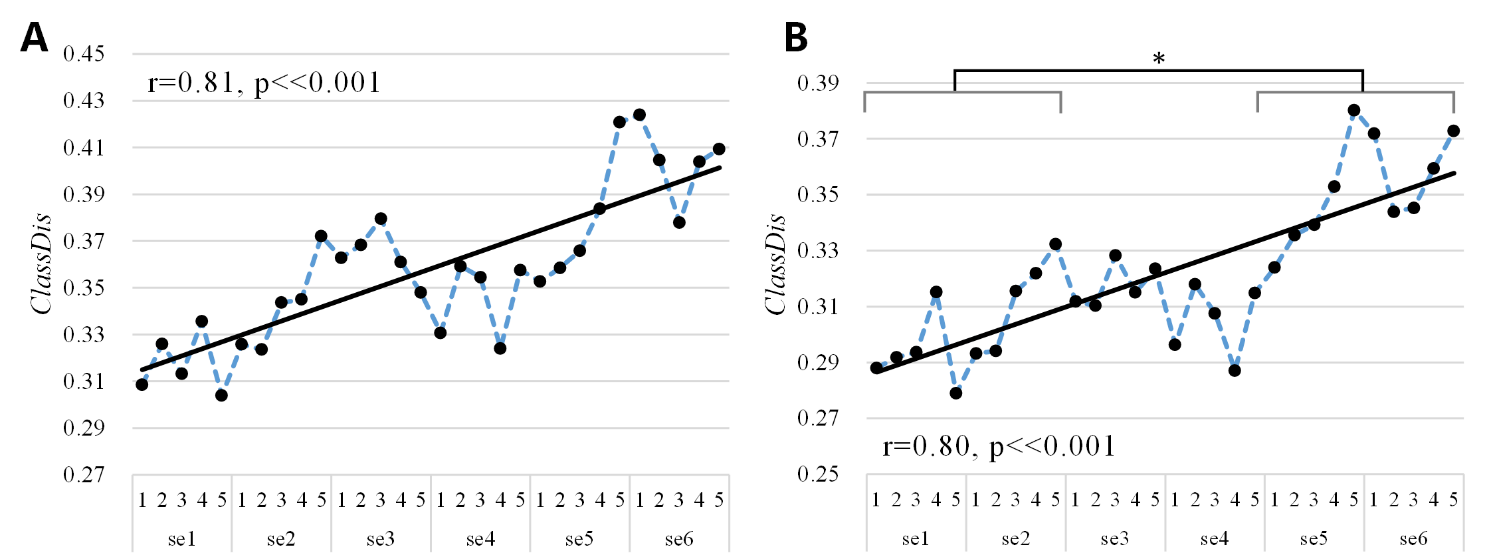


**Supplementary Figure 2.** The average **(A)** "broadband" and **(B)** “subject-specific” learning curve of *ClassDis* for two MI tasks across 6 sessions among ten subjects. The corresponding linear fits and Pearson correlation coefficients demonstrate training effects. The first and last session (day) of *ClassDis* were tested for significant differences using the custom complex contrast of the repeated measures ANOVA model, $*p<0.05$.

**
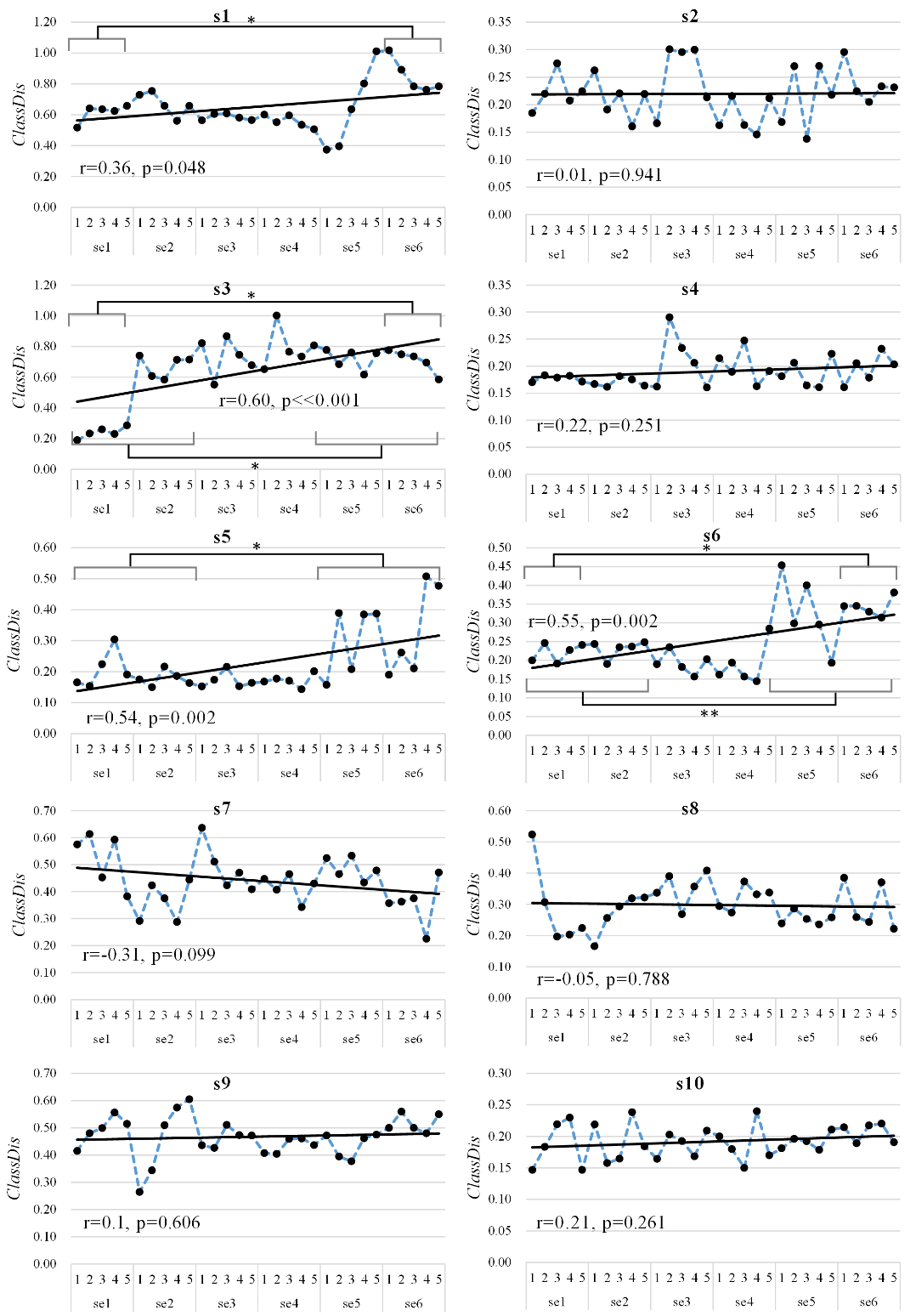
**

**Supplementary Figure 3.** The “broadband” learning curve of *ClassDis* for two MI tasks throughout training for each subject. The corresponding linear fits and Pearson correlation coefficients demonstrate training effects. The first and last session (day) of *ClassDis* were tested for significant differences using two-sided Wilcoxon ranksum tests, $*p<0.05$.

**
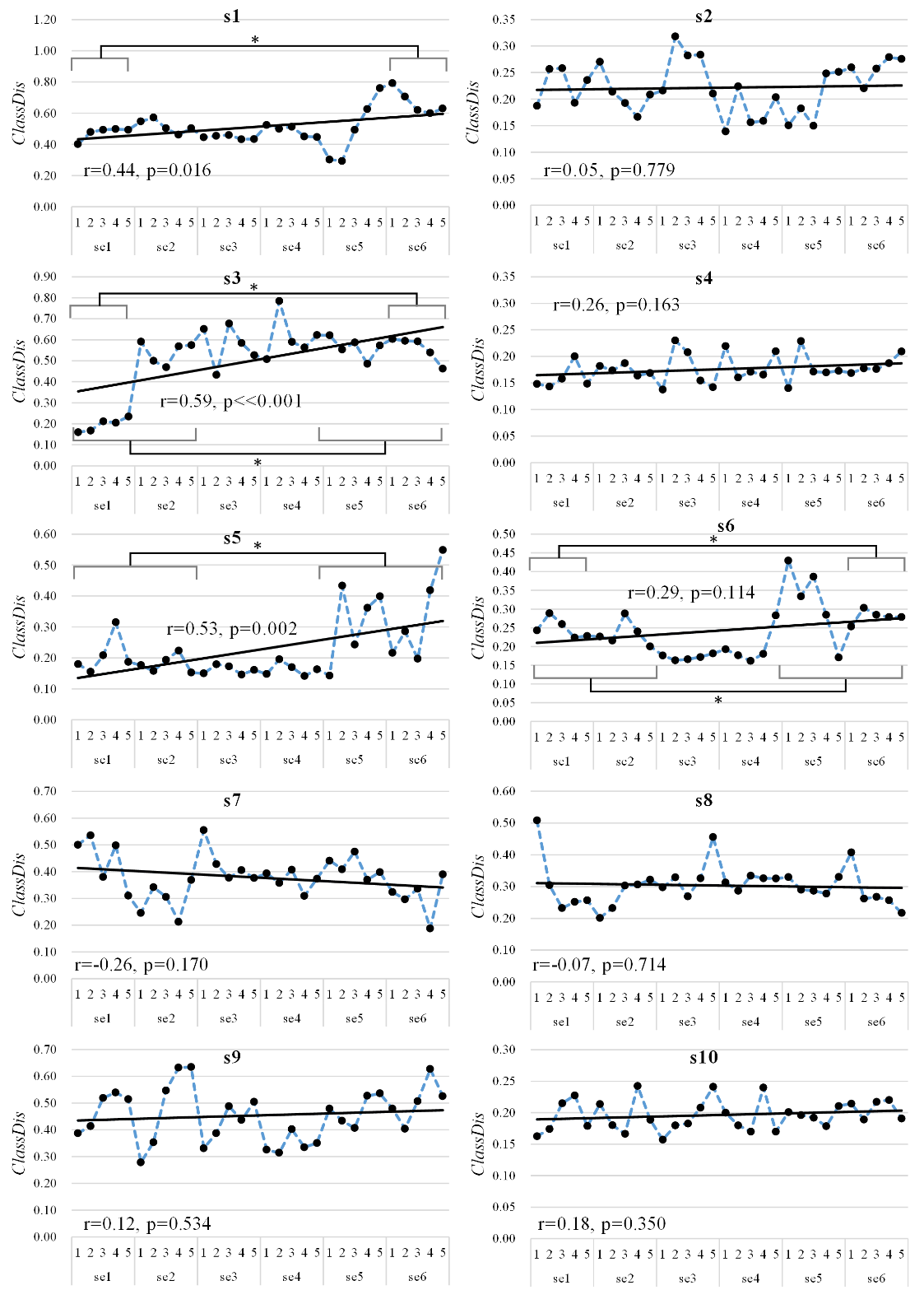
**

**Supplementary Figure 4.** The “subject-specific” learning curve of *ClassDis* for two MI tasks throughout training for each subject. The corresponding linear fits and Pearson correlation coefficients demonstrate training effects. The first and last session (day) of *ClassDis* were tested for significant differences using two-sided Wilcoxon ranksum tests, $*p<0.05$.

**
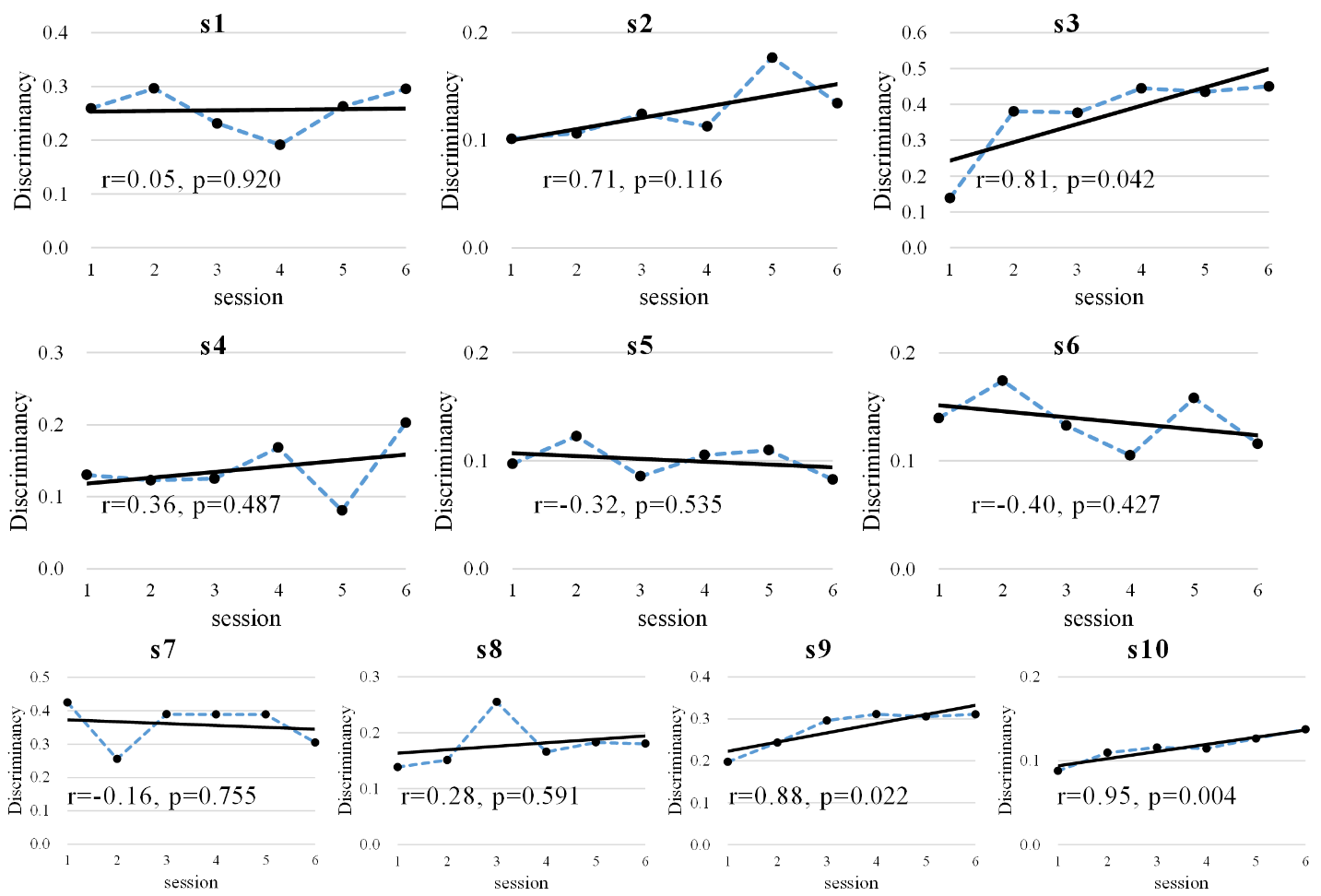
**

**Supplementary Figure 5.** The “actual, online” learning curve of EEG feature discriminancy for 10 subjects. The corresponding linear fits and Pearson correlation coefficients are reported to indicate training effects.

**
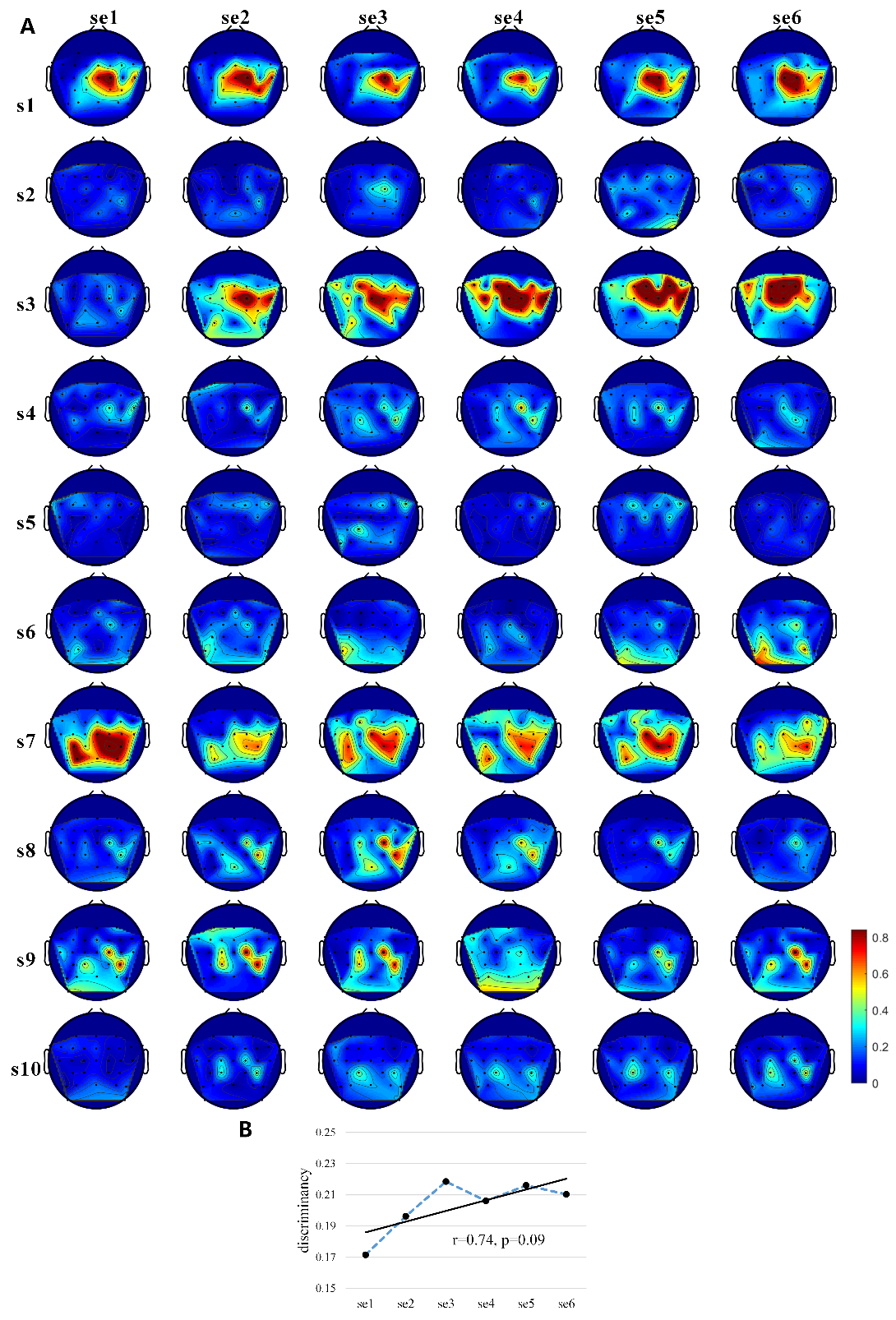
**

**Supplementary Figure 6.** **Discriminancy of EEG feature.** **(A)** The “broadband” topographic maps of discriminancy per training session on the 30 EEG channels locations over the sensorimotor cortex. Red indicates high discriminancy between lefthand-MI and righthand-MI tasks employed by 10 subjects. The discriminancy of each channel is quantified as the Fisher score of power spectral density distributions of EEG for two MI tasks in the “broadband” frequency band within each session. **(B)** Average “broadband” learning curve of EEG feature discriminancy for 10 subjects. Discriminancy value of each session calculated by averaging Fisher scores among all 30 channels. Corresponding linear fits and Pearson correlation coefficients are reported to indicate training effects.

**
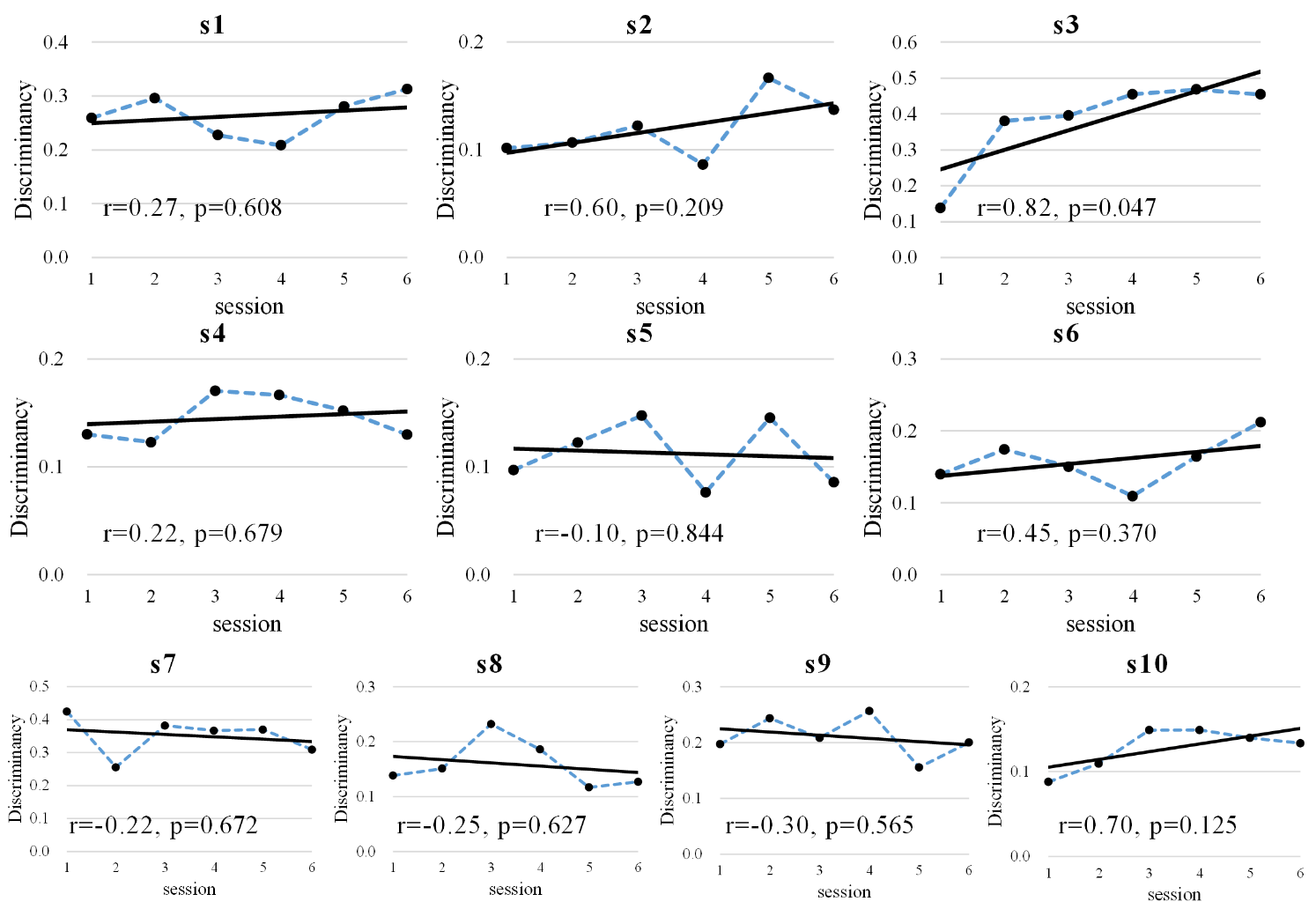
Supplementary Figure 7.** The “broadband” learning curve of EEG feature discriminancy for 10 subjects. The discriminancy value of each session is calculated by averaging the Fisher score among all 30 channels. The corresponding linear fits and Pearson correlation coefficients are reported to indicate training effects.

**
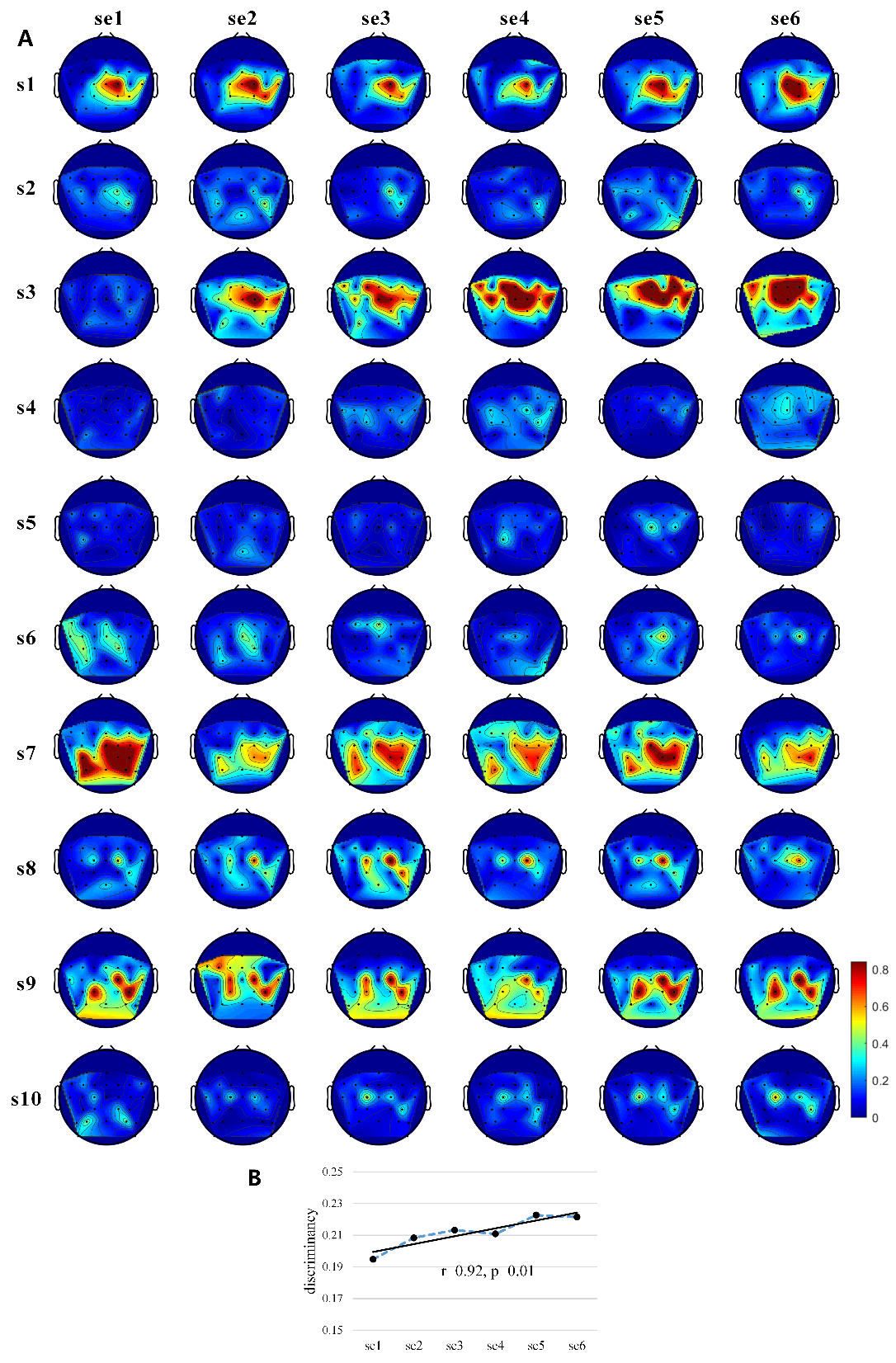
**

**Supplementary Figure 8. Discriminancy of EEG feature. (A)**The “subject-specific” topographic maps of discriminancy per training session on the 30 EEG channels locations over the sensorimotor cortex. Red indicates high discriminancy between lefthand-MI and righthand-MI tasks employed by 10 subjects. The discriminancy of each channel is quantified as the Fisher score of power spectral density distributions of EEG for two MI tasks in the “subject-specific” frequency band within each session. **(B)** Average “subject-specific” learning curve of EEG feature discriminancy for 10 subjects. Discriminancy value of each session calculated by averaging Fisher scores among all 30 channels. Corresponding linear fits and Pearson correlation coefficients are reported to indicate training effects.

**
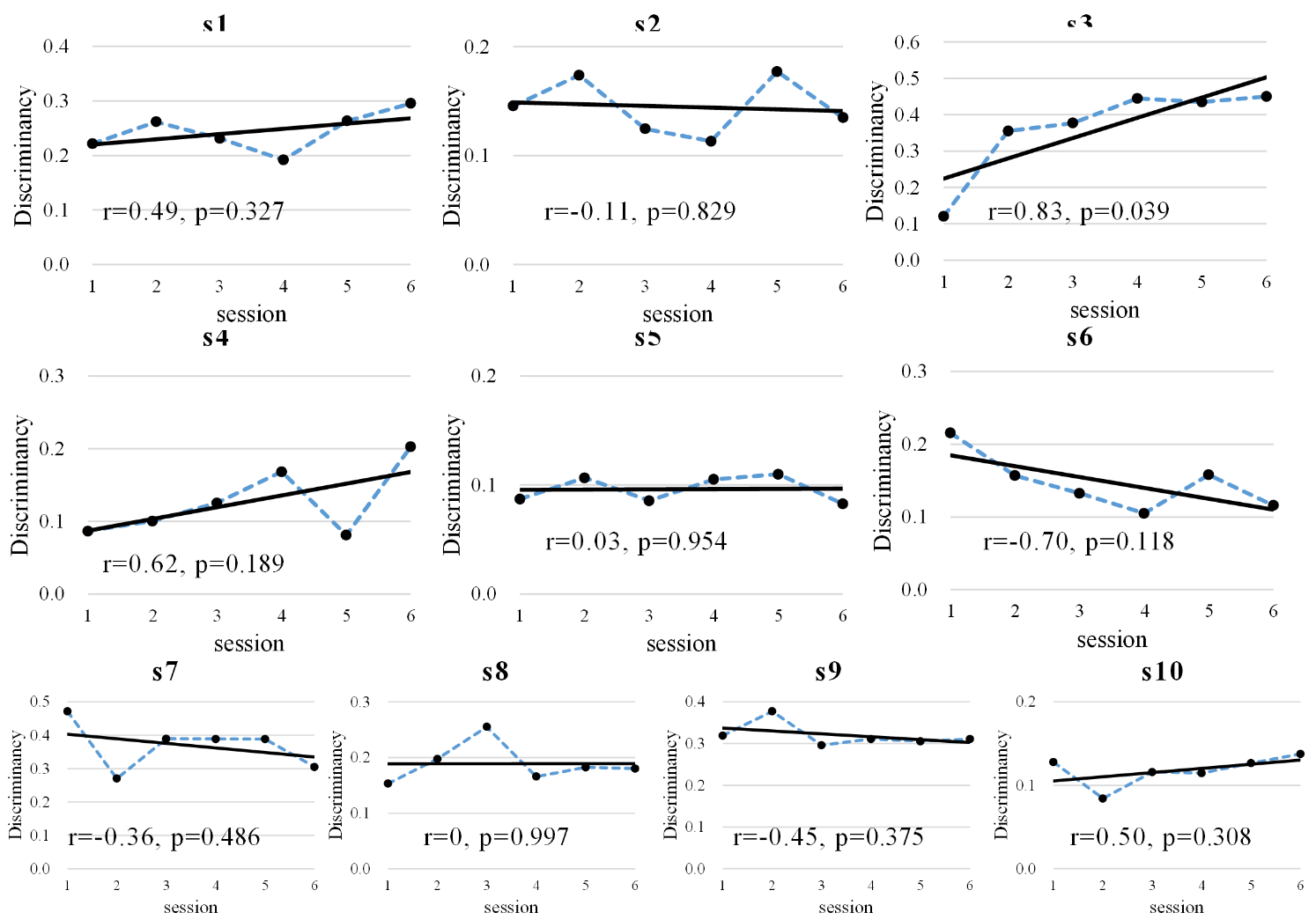
**

**Supplementary Figure 9.** The “subject-specific” learning curve of EEG feature discriminancy for 10 subjects. The discriminancy value of each session is calculated by averaging the Fisher score among all 30 channels. The corresponding linear fits and Pearson correlation coefficients are reported to indicate training effects.

**
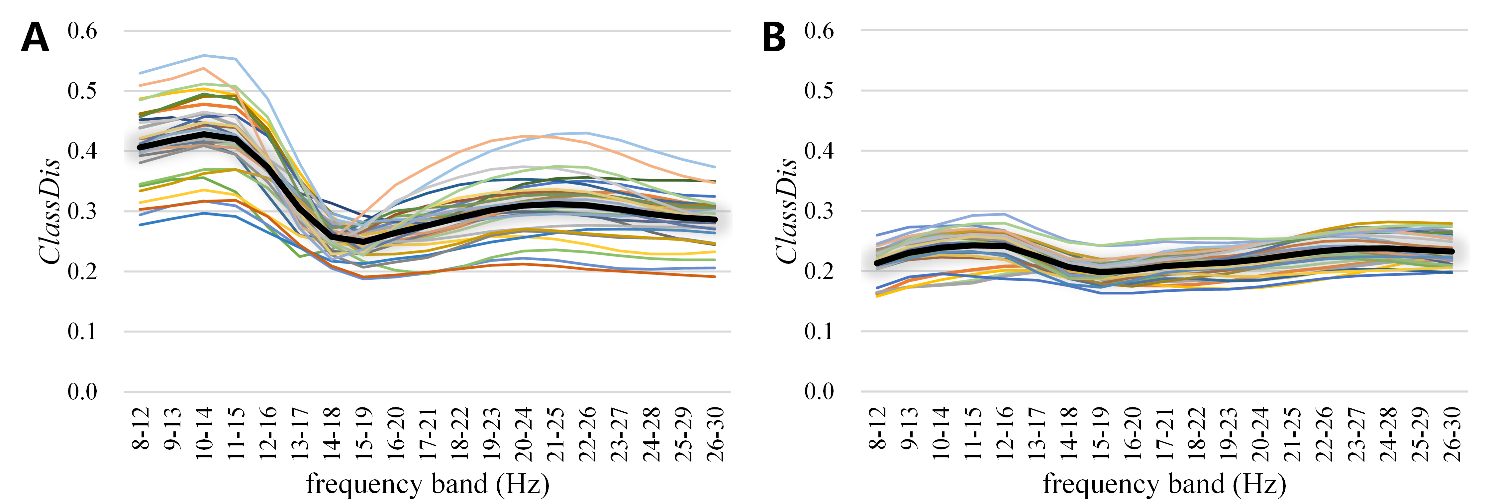
**

**Supplementary Figure 10****.** ${ClassDis}_{f}$ for 19 frequency bands. **(A)** Average of subjects with good MI-BCI skill and **(B)** average of subjects with low skill. Thirty colorful lines represent ${ClassDis}_{f}$ for 30 runs; thick black line represents the average among them.

**
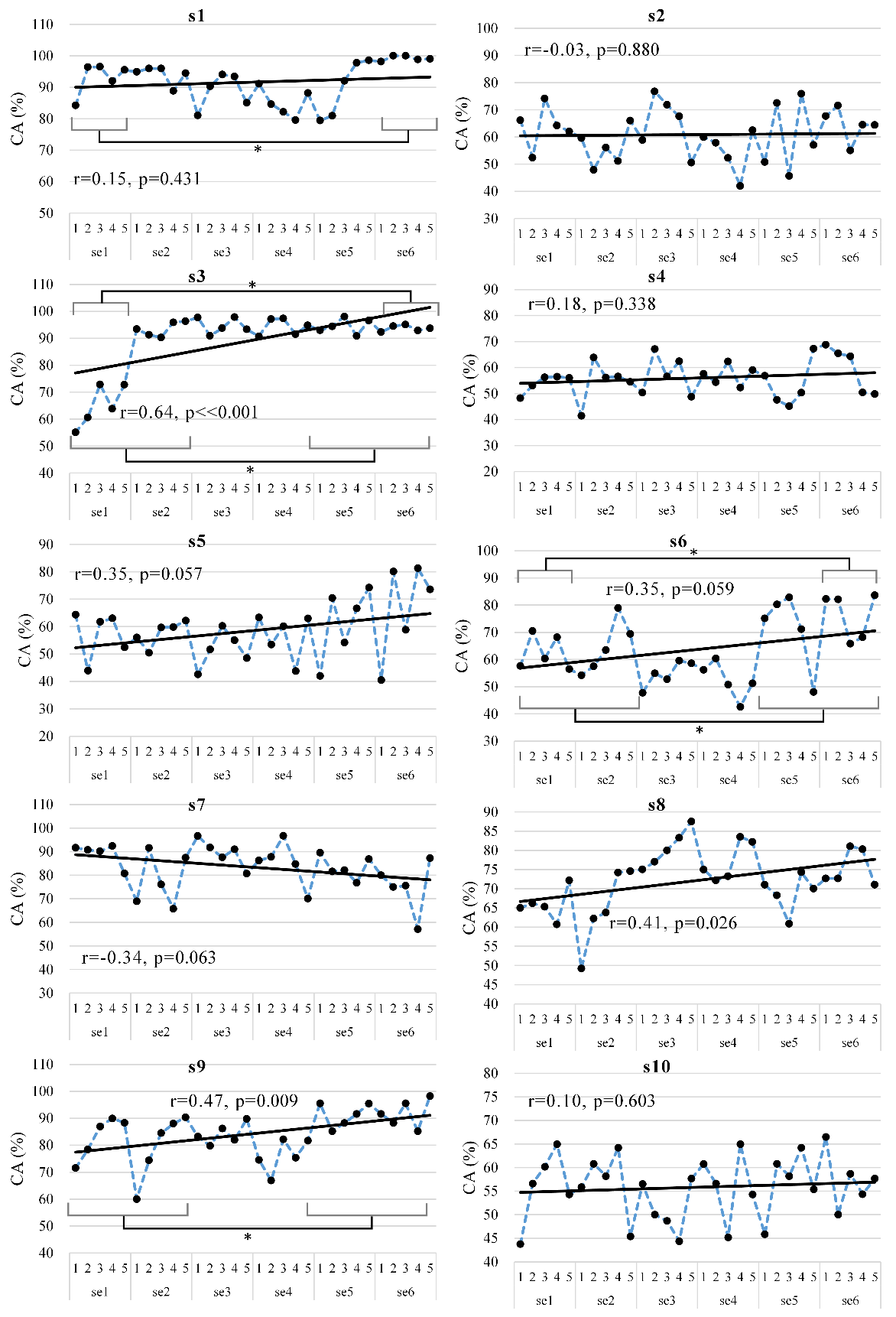
**

**Supplementary Figure 11.** The “broadband” learning curve of classification accuracy for two MI tasks throughout training for each subject. The corresponding linear fits and Pearson correlation coefficients demonstrate training effects. The first and last session (day) of *ClassDis* were tested for significant differences using two-sided Wilcoxon ranksum tests, $*p<0.05$.

## Supplementary Videos

**Supplementary Video 1.** The online video for the feedback training experiment.

**Supplementary Video 2.** The one-by-one appearance of feedback points by diffusion map projection during a run for s1 (good performer).

**Supplementary Video 3.** The one-by-one appearance of feedback points by diffusion map projection during a run for s8 (lower performer).
